# New mechanisms of normoxic and hypoxic cGMP signalling mediated by the nitrite anion

**DOI:** 10.1186/2050-6511-14-S1-O20

**Published:** 2013-08-29

**Authors:** Hunter Champion

**Affiliations:** 1 University of Pittsburgh, Pittsburgh, PA 15213, USA

## Backgound

Chronic treatment with sodium nitrite has been shown to be efficacious in preclinical models of pulmonary arterial hypertension (PAH). The present study was designed to test the acute hemodynamic effects and safety of inhaled nitrite in patients with PAH (treatment naïve or on background therapy). Details: http://www.clinicaltrials.gov NCT01431313 METHODS: Inhaled nitrite was delivered at incremental doses (45 and 90 mg) to WHO group I and WHO Group III PAH patients undergoing clinical right heart catheterization for the diagnosis or management of PAH. In addition to clinical measures of safety and treatment response, novel hemodynamic parameters of RV function were acquired by both micromanometry and echocardiography.

## Results

Inhaled nitrite was well tolerated by all subjects (n=10) at both the 45mg and 90 doses in treatment naïve and patients on background therapy (mean systemic blood pressure and systemic vascular resistance were not altered at either dose (NS; *P*>0.05) including patients on background PDE5A inhibitor therapy (n=8). Inhaled nitrite lowered mean pulmonary arterial pressure (mPAP) and pulmonary vascular resistance (PVR) in a dose-dependent manner. Inhaled nitrite 45mg and 90 mg doses lowered mPAP by 9.6+0.6% and 12.4+2.2 (*P*<0.0001) and PVR by 12.4+5.3% and 19.3+5.3% (*P*=0.006), respectively. Interestingly, inhaled nitrite resulted in a marked lowering of right atrial pressure (*P*<0.001) and pulmonary capillary wedge pressure (*P*<0.001). When coupled with the observation of improvements in dP/dt_min_, these data suggest that inhaled nitrite may exert a novel lusitropic ventricular effect. Nitrite inhalation resulted in a significant increase in plasma cGMP concentrations.

## Conclusion

This preliminary analysis of an open-label study of inhaled nitrite in patients with PAH provide evidence for tolerability of the drug in treatment naïve and patients on background therapy (including PDE5A inhibitors). Moreover, these data demonstrate that inhaled nitrite induces a dose-dependent reduction in mPAP and PVR in patients with PAH.

